# Plasminflammation—An Emerging Pathway to Bradykinin Production

**DOI:** 10.3389/fimmu.2019.02046

**Published:** 2019-08-27

**Authors:** Coen Maas

**Affiliations:** Department of Clinical Chemistry and Haematology, University Medical Center Utrecht, Utrecht University, Utrecht, Netherlands

**Keywords:** plasmin, bradykinin, angioedema, contact system, factor XII

## Abstract

Plasminogen activation is essential for fibrinolysis—the breakdown of fibrin polymers in blood clots. Besides this important function, plasminogen activation participates in a wide variety of inflammatory conditions. One of these conditions is hereditary angioedema (HAE), a rare disease with characteristic attacks of aggressive tissue swelling due to unregulated production and activity of the inflammatory mediator bradykinin. Plasmin was already implicated in this disease decades ago, but a series of recent discoveries have made it clear that plasmin actively contributes to this pathology. Collective evidence points toward an axis in which the plasminogen activation system and the contact system (which produces bradykinin) are mechanistically coupled. This is amongst others supported by findings in subtypes of HAE that are caused by gain-of-function mutations in the genes that respectively encode factor XII or plasminogen, as well as clinical experience with the antifibrinolytic agents in HAE. The concept of a link between plasminogen activation and the contact system helps us to explain the inflammatory side effects of fibrinolytic therapy, presenting as angioedema or tissue edema. Furthermore, these observations motivate the development and characterization of therapeutic agents that disconnect plasminogen activation from bradykinin production.

## Introduction

### Plasminogen Activation: More Than “Just” Fibrinolysis?

The main purpose of plasminogen activation is to break down fibrin in blood clots. Hereto, tPA (tissue-type plasminogen activator) from endothelial cells binds to fibrin. Next, tPA activates adjacent plasminogen molecules which cleave fibrin. Alternatively, the urokinase system can generate plasmin. Hereto, uPA (urokinase type-plasminogen activator) binds to a dedicated receptor (uPAR) on activated endothelial cells, circulating cells (e.g., monocytes), and tissue-resident cells. Interestingly, uPAR expression can take place in situations that are unrelated to fibrinolysis. For example, endothelial cells can sense hypoxia and express uPAR in response (1). Alternatively, there is a proposed role for VEGF-induced uPAR expression in angiogenesis (2). Furthermore, uPAR expression is strongly upregulated at sites of inflammation (3), indicating that plasmin has roles beyond fibrinolysis.

### The Contact System: More Than “Just” Coagulation?

The contact system consists of factor XII (FXII), plasma prekallikrein (PK) and high-molecular weight kininogen (HK). These factors assemble on (negatively) charged particles and polymers to generate enzymatic activity. The contact system owes its name to the clotting response that follows when blood contacts surface materials, such as the diagnostic coagulation reagent kaolin. Since its discovery, it has become clear that the contact system is a driving force behind thrombosis and hemoincompatibility (4). Remarkably, deficiency in contact factors does not translate into bleeding disorders (5–7), suggesting that a function beyond hemostasis justifies the existence of this enzyme system.

During contact activation, FXII and PK activate each other through reciprocal cleavage into FXIIa and PKa. C1-inhibitor (C1-INH) controls both enzymes. HK is essential to this reaction, as it connects PK to the surface. Truncation of full-length FXIIa (αFXIIa) by PKa into βFXIIa eliminates its clotting potential. However, βFXIIa remains an excellent fluid-phase PK activator (8). Most information on contact system activation *in vitro* strongly suggests that FXII activation requires a surface. However, clinical observations point toward to a complimentary mechanism for FXII activation, dissimilar from classical surface-bound contact activation.

### Links Between Plasminogen Activation and Contact Activation (Figure 1)

FXII is strikingly homologous to tPA (Figure 2). They both contain kringle domains, epidermal growth factor-like domains, as well as fibronectin-type I domains. To a certain extent, FXII and tPA are biochemically inter-exchangeable. In 1972, it was reported that FXIIa can act as a plasminogen activator (9). More recently, it was reported that fibrin-bound polyphosphate polymers amplify this reaction (10). There is some clinical evidence supporting the role of FXIIa as plasminogen activator: FXII-deficient human subjects have a lowered capacity for plasminogen activation in response to systemically administered desmopressin (activates endothelial cells) (11). Future studies are needed to disentangle the seemingly conflicting roles of FXII as clotting factor and plasminogen activator.

**Figure 1:**
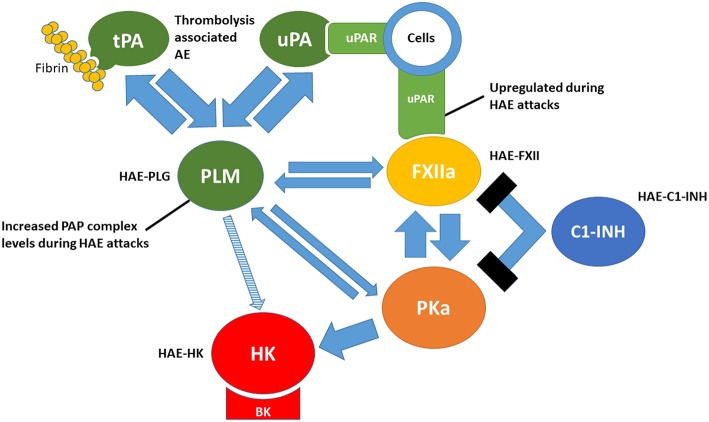
Links between the plasminogen activation and contact activation. tPA, tissue-type plasminogen activator; uPA, urokinase-type plasminogen activator; uPAR, urokinase-type plasminogen activator receptor; PLM, plasmin; FXIIa, activated factor XII; PKa, plasma kallikrein; HK, high molecular-weight kininogen; BK, bradykinin; C1-INH, C1 inhibitor. PAP, plasmin-α2-antiplasmin. HAE-PLG, HAE-FXII, HAE-C1-INH and HAE-HK represent forms of hereditary angioedema related to gain-offunction mutations in each factor.

**Figure 2:**
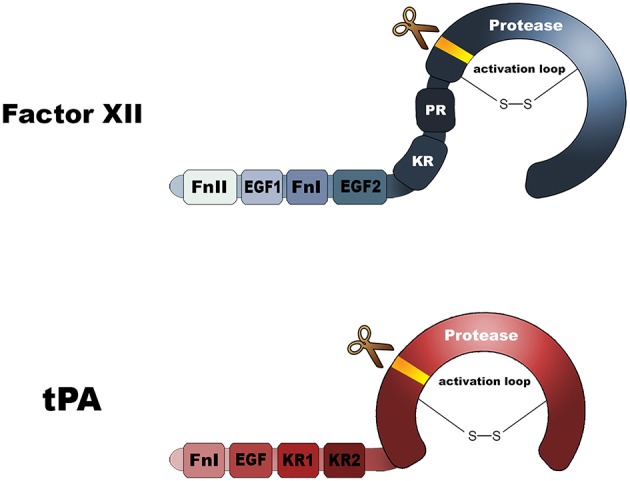
Domain architectures of factor XII and tissue-type plasminogen activator. FnI, Fibronectin type I domain; FnII, Fibronectin type II domain; EGF, epidermal growth factor-like domain; KR, Kringle. Both molecules contain a protease domain that becomes active after molecular scission, resulting in an two-chain disulfide-linked molecule.

Conversely, plasmin can also act as a FXII activator. In 1971, it was identified that plasmin can activate FXII into FXIIa (12). We recently confirmed this (13). However, the original biochemical observation remained without consequence for decades. This is largely attributable to the fact that PKa is a much more competent FXII activator than plasmin is. Plasmin can also act as a direct and reciprocal PK activator, and accelerates bradykinin release from HK (14). Together, these biochemical links between plasminogen activation and the contact system make it attractive to speculate that in the very early stages of *in vivo* contact activation, when PKa has yet to become activated; plasmin has an initiating role. Lessons from human pathology, such as hereditary angioedema and neuroinflammation, suggest that this might be the case.

## Hereditary Angioedema

### C1 Inhibitor Deficiency

Hereditary angioedema (HAE) is a rare disease with characteristic swelling of the deep skin and mucosa caused by local vascular leakage. The onset of tissue swelling attacks is highly unpredictable, but reported triggers include physical exertion, mental stress, mechanical trauma and infections (15). Experienced patients report prodromal symptoms; telltale signs that an attack is imminent (16). Most notably, these patients may have erythema marginatum, a nonpruritic rash that presents gradually (17) and can become clinically apparent very early in life (18).

HAE was first clinically identified in 1888 (19), and connected to C1 inhibitor deficiency in 1963 (20). The associated disease is now called HAE-C1-INH (OMIM # 106100) and affects 1:50,000 people. To date, 488 mutations have been identified that cause HAE-C1INH (http://www.hgmd.cf.ac.uk). Some of these are *de novo* mutations (21). There are two subtypes: quantitative deficiency (type I) and qualitative deficiency (type II). Interestingly, in some heterozygous type I HAE-C1-INH carriers, C1-INH expression levels that are far below the expected 50%. For a subset of these mutations, there is an explanation: the mutation causes C1-INH to form intracellular aggregates, which incorporate “healthy” wild type C1-INH as well and prevent secretion (22).

In search for the disease mediator in HAE, it was initially thought that unregulated complement activation caused the tissue swelling attacks. Around the same time, it was suggested that PKa activity was involved in the disease phenotype (23). However, it took decades to directly identify bradykinin as central mediator (24, 25). At present, a variety of therapeutic strategies are available that have the aim to reduce bradykinin production, including C1-INH replacement therapy, or monoclonal antibodies and oral therapies that target and control PKa (26, 27).

There are two receptors for bradykinin (and Lys-bradykinin, which is generated by tissue kallikreins): the kinin B2- and B1 receptors (kB2R and kB1R, respectively). Whereas kB2R is constitutively present on the vascular endothelium, kB1R is expressed by cells at sites of infection and inflammation. KB2R mainly recognizes full-length (lys-)bradykinin. Removal of the C-terminal lysine from (lys-)bradykinin by soluble carboxypeptidase N or membrane-bound carboxypeptidase M, generates a sequence that is preferred by kB1R. Endothelial kB2R activation induces cytoskeletal rearrangements, uncoupling of tight junctions and triggers NO production, which instructs underlying endothelial cells to relax. A direct, small molecule inhibitor of kB2R has therapeutic value during acute attacks, showing that the interaction between full length (lys-)bradykinin and kB2R is key to HAE pathology (28).

The mechanism behind the localized presentation of the clinical symptoms in HAE is of high interest. It has been proposed that a currently unidentified trigger incites systemic contact activation. Excessive localized vascular leakage is a consequence of locally increased cellular kB1R presentation (29). Indeed, there is evidence for activation products of contact activation during (30), and before the onset of attacks (31). On the other hand, the localized detection of bradykinin at the site swelling (32), together with its limited circulating half-life suggest that bradykinin production is a localized process (33).

## Plasminflammation: Plasmin in HAE-C1-INH

Plasminogen activation is seen during attacks of HAE-C1-INH (34–38). It remains a question what triggers and drives this plasminogen activation. It can be speculated that it is secondary to bradykinin-driven endothelial cell activation, which is accompanied by release of plasminogen activators (39) and depletion of PAI-1 (40). Alternatively, marker for coagulation are also increased in HAE patients: thrombin-antithrombin complex levels, prothrombin fragment 1+2 and D-dimer levels are all increased (36, 41). This suggests that coagulation is first triggered when plasma leaks into the extravascular space, which in turn triggers plasmin formation. However, a study in HAE patient shows that C1-INH replacement therapy lowers F1+2 levels, while fibrinolytic parameters remain elevated (42). This suggests that plasminogen activation during HAE-C1INH attacks is unrelated to its role in “normal” fibrinolysis.

Interestingly, circulating peripheral blood cells of C1-INH deficient patients express increased amounts uPAR during swelling attacks (43). Furthermore, these patients benefit from prophylactic treatment with the antifibrinolytic agent tranexamic acid, which reduces both the frequency and severity of HAE attacks (44). Finally, careful *in vitro* studies show that C1-INH-deficient plasma generates excessive amounts of bradykinin when tPA is added (45). This occurs in a FXII and PKa-dependent manner. These combined observations suggests that plasmin has an active role in the pathogenesis of HAE-C1-INH.

### Hereditary Angioedema With Normal C1-INH Activity

Not all forms of HAE are attributable to C1-INH deficiency: there are cases in which patient families experience tissue swelling attacks while both levels and activity of C1-INH are normal (46). These together are classified as type III HAE (OMIM # 610618). So far, there are four individual forms (47). Below, I will highlight three of those that may be linked:

#### HAE-FXII

This form is caused by mutations in the F12 gene (encodes FXII). It was first identified in 2006 in a genetic study under HAE patients with normal C1-INH activity (48), and connected to increased spontaneous FXII activity in plasma (49). Since then, five separate mutations have been found that give the same phenotype (50). These patients are often female, and respond well to kB2R antagonism, as well as tranexamic acid. This indicates that, also in these patients, bradykinin is the responsible disease mediator and that plasmin contributes to pathogenesis. On protein sequence level, these mutations are all located the proline-rich region of FXII. This unstructured and flexible sequence connects the surface-binding domains of FXII to its protease domain (51). Investigations into the underlying disease mechanisms showed us that mutations c.1032C>A (results in T309K in the mature protein) and c.1032C>G (results in T309R in the mature protein) both eliminate an O-linked glycosylation site from the proline-rich region (52). This enhances activation by the anionic polymer dextran sulfate. However, C1-INH inhibits both these FXIIa mutants with the same efficiency as it can inhibit wild type FXIIa. In other words, these mutants are not resistant against inhibition. In further studies, we identified that the replacement of the threonine (T) residue at position 309 with a positively charged arginine (R) or lysine (K) residue introduces putative cleavage sites for trypsin-like serine proteases (13). The same holds true for mutation c.971_1018 + 24del72 (this replaces an existing sequence with a new one that contains 5 arginines). Although PKa is essential to the activation and subsequent processing of normal wild type FXII, we found that it did not cleave at the putative newly introduced cleavage sites. We next considered plasmin as a candidate enzyme as it A) has the ability to directly activate FXII; B) is active in HAE-C1-INH and C) tranexamic acid has value for HAE-FXII (53). We found that plasmin indeed cleaves these three pathogenic forms of FXII at the mutated positions, separating the surface binding domains from the protease domain (13). The resulting fragment is in many aspects similar to βFXIIa, but in this case, it has not yet been activated (i.e., it should be called βFXII). However, this truncating event leads FXII to expose its activation loop, leaving it highly sensitive to activation by PKa or plasmin in solution (54). In similar manner, others have more recently found that FXII mutants T309K and T309R can also be truncated by thrombin and FXIa (55). This sets the stage for swelling attacks after injury in HAE-FXII patients. There are two more mutations in FXII that cause HAE: c.892_909dup (duplicates residues 279–284 in mature FXII) and c.1027G>C (A324P in mature FXII). We found that these are not susceptible to truncation by plasmin (unpublished findings) and it currently remains unclear how these mutations cause pathology.

#### HAE-PLG

Very recently, Bork et al. identified a mutation in the plasminogen gene through wholeexome sequencing studies in German patient families that have HAE with normal C1-INH activity. The mutation c.9886A>G causes an amino acid substitution in kringle 3 (K311E; mature protein) (56). The interesting thing about this mutation is that it should restore the lysine-binding properties of kringle 3 that are normally lacking in this human plasminogen kringle (57). It is of high interest and clinical relevance to elucidate how this mutation translates into the seemingly selective clinical phenotype of recurrent angioedema, rather than a bleeding diathesis (this is the picture of hyperfibrinolysis). Conversely, it is interesting that α2-antiplasmin deficiency (hyperfibrinolysis) does not appear to be accompanied by attacks of tissue edema.

Since the first report on HAE-PLG, the very same mutation has been found in patients from Japan (58), Bulgaria, Spain, Greece (59) and another German patient family (60). Germenis et al. also pointed out that the mutation may not cause disease purely in a stand-alone manner: several patients in their study had additional polymorphisms in the plasminogen gene or alternatively in enzymes that are involved in bradykinin metabolism (59). Not completely surprisingly, these patients respond well to prophylactic treatment with tranexamic acid, indicating that lysine-dependent target engagement of mutant plasminogen is a critical step in the disease mechanism. More importantly, HAE-PLG patients that experience acute attacks respond well to the kB2R antagonist icatibant, suggesting that bradykinin is an important disease mediator during swelling attacks (60).

#### HAE-HK

Even more recently, a new form of HAE with normal C1-INH was identified in a family with a mutation in the KNG1 gene (c.1136T>A; p.Met379Lys). This gene encodes HK, as well as the splice variant low-molecular weight kininogen (LK). The mutation is located in close vicinity to the physiological N-terminal cleavage sites that normally mediates (lys-)bradykinin liberation from its precursor protein, and therefore highly likely to change the release kinetics of bradykinin-based vasoactive peptides. This exciting discovery leads to many questions. Does this mutation selectively affects the release of bradykinin by PKa, or the release of lys-bradykinin by tissue kallikreins? Does the sequence Lys-lys-bradykinin (or its des-Arg metabolite) react normally with kB2R and kB1R?

## Implications For The Brain

Tissue swelling and edema formation are general features of both chronic and acute inflammation. In a surprisingly broad and growing spectrum of diseases beyond HAE, bradykinin has been implicated as a disease mediator. For example, FXII-dependent bradykinin production is held responsible for impairment of the blood-brain barrier in Alzheimer's disease. It has been repeatedly found that aggregated amyloid β peptide can directly trigger FXII, leading to PKa activity and targeting of this inflammatory pathway has therapeutic value in models for this disease (61–63). Interestingly, experimental knockdown of C1-INH expression in mouse studies recapitulates many of these neurological features without the apparent involvement of amyloid β peptide. This suggests that proper control over bradykinin production is essential for a healthy blood-brain barrier in general (64), and might relate to the signs of depression that are reported in HAE patients (65, 66).

So how does plasmin fit into this picture? Targeting plasminogen expression in mouse models for Alzheimer's disease attenuates disease progression similar to knockdown of FXII (67). In similar manner, LPS-triggered neuroinflammation is attenuated in mice that lack plasminogen or tPA (68). The other way around, experimental induction of sustained hyperfibrinolysis by system overexpression of plasminogen activators in mice leads to increased permeability of the bloodbrain barrier (69). This is attributable to plasmin-driven bradykinin production, fitting with the clinical observation that bradykinin-driven angioedema is a rare (1–5%) side-effect of thrombolytic therapy (70). However, brain edema after thrombolytic therapy is much more common and generally considered an unavoidable treatment-associated evil. Interestingly, preclinical studies point out that this phenomenon is actually caused by PKa-driven bradykinin production (71).

## Therapeutic Targeting Of Plasminflammation

Evidently, plasmin-mediated breakdown of blood clots is required for a healthy vasculature; hypofibrinolysis is a risk factor for venous thromboembolism (72). Although prophylactic treatment of HAE patients with antifibrinolytic agents is well-tolerated, human plasminogen deficiency is associated with development of ligneous conjunctivitis (73). This suggests that generalized neutralization of plasminogen activation might have adverse effects. Considering that the urokinase system can mediate plasminogen activation in a fibrin-independent manner, it is attractive to speculate that selective targeting of this axis has benefit in repressing excessive plasmin-dependent bradykinin formation, while tPA remains available for physiological fibrinolysis. Although this a possibility, how about simply targeting the contact factors?

*In vivo* studies in mouse models, human deficiencies and drug trials have shown us that targeting the contact system is generally safe. However, it should be remembered that there is a proposed role for FXII-dependent coagulation in thrombus stabilization (74). For blockade of plasminflammation, it would be needed to selectively inhibit the molecular interactions between plasmin(ogen) and FXII or other contact factors. Identification of these interaction sites is of high interest, as these can be used for development of agents (e.g., monoclonal antibodies) that selectively neutralize plasminflammation, while leaving other physiological functions of the involved systems intact.

## Conclusion

When combining insights from biochemical studies and clinical observations in rare diseases, it becomes clear that plasminogen activation and the plasma contact system are functionally intertwined. Although the role of FXIIa as plasminogen activator was discovered first, the role of plasmin as contact activator is clinically more apparent. In many states of pathology where FXII-driven bradykinin production contributes to inflammation, the endogenous FXII activator has yet to be identified. However, more often than not, plasmin is present and ready. Development of strategies to uncouple fibrinolysis from plasmin-triggered bradykinin production should have value for treatment of inflammatory conditions.

## Author Contributions

The author confirms being the sole contributor of this work and has approved it for publication.

### Conflict of Interest Statement

The author declares that the research was conducted in the absence of any commercial or financial relationships that could be construed as a potential conflict of interest.
